# Decreased expression of p57^KIP2^mRNA in human bladder cancer

**DOI:** 10.1054/bjoc.2000.1298

**Published:** 2000-08-16

**Authors:** M Oya, W A Schulz

**Affiliations:** Urologische Klinik, Heinrich Heine Universität, Moorenstrasse 5, Düsseldorf, D-40225, Germany

**Keywords:** chromosome 11p15.5, IGF-II, H19, quantitative RT-PCR, LOH analysis

## Abstract

To identify targets of genetic and epigenetic alterations on chromosome 11p15.5 in human bladder cancer, expression of the imprinted *KIP2*, *IGF2* and *H19* genes was studied by quantitative RT-PCR in 24 paired samples of urothelial carcinomas and morphologically normal mucosa obtained by cystectomy, and in bladder carcinoma cell lines. The most frequent alteration in tumour tissue was decreased expression of *KIP2* identified in 9/24 (37%) specimens. Decreased *IGF2* and *H19* mRNA levels were found in five (21%) and three (13%) tumours, respectively. One tumour each overexpressed *IGF2* and *H19*. Loss of *H19* expression was only found associated with loss of *KIP2* expression, whereas decreased expression of *IGF2* mRNA occurred independently. Almost all bladder carcinoma cell lines showed significant changes in the expression of at least one gene with diminished expression of *KIP2* mRNA as the most frequent alteration. *IGF2* mRNA levels were diminished in several lines, but increased in others. The *KIP2* gene could be an important target of genetic and epigenetic alterations in bladder cancer affecting the maternal chromosome 11p15.5. However, reminiscent of the situation in Wilms’ tumours, expression of the *IGF2* gene on the paternal chromosome can also be disturbed in bladder cancers. © 2000 Cancer Research Campaign


*British Journal of Cancer* (2000) **83**(5), 626–631
© 2000 Cancer Research Campaign
doi: 10.1054/ bjoc.2000.1298, available online at http://www.idealibrary.com on IDEAL®

### Decreased expression of p57$^{KIP2}$ mRNA in human bladder cancer

**M Oya and WA Schulz**

Urologische Klinik, Heinrich Heine Universität, Moorenstrasse 5, D-40225 Düsseldorf, Germany

**Summary** To identify targets of genetic and epigenetic alterations on chromosome 11p15.5 in human bladder cancer, expression of the imprinted *KIP2*, *IGF2* and *H19* genes was studied by quantitative RT-PCR in 24 paired samples of urothelial carcinomas and morphologically normal mucosa obtained by cystectomy, and in bladder carcinoma cell lines. The most frequent alteration in tumour tissue was decreased expression of *KIP2* identified in 9/24 (37%) specimens. Decreased *IGF2* and *H19* mRNA levels were found in five (21%) and three (13%) tumours, respectively. One tumour each overexpressed *IGF2* and *H19*. Loss of *H19* expression was only found associated with loss of *KIP2* expression, whereas decreased expression of *IGF2* mRNA occurred independently. Almost all bladder carcinoma cell lines showed significant changes in the expression of at least one gene with diminished expression of *KIP2* mRNA as the most frequent alteration. *IGF2* mRNA levels were diminished in several lines, but increased in others. The *KIP2* gene could be an important target of genetic and epigenetic alterations in bladder cancer affecting the maternal chromosome 11p15.5. However, reminiscent of the situation in Wilms' tumours, expression of the *IGF2* gene on the paternal chromosome can also be disturbed in bladder cancers. © 2000 Cancer Research Campaign

**Keywords:** chromosome 11p15.5; IGF-II; H19; quantitative RT-PCR; LOH analysis

Cytogenetic and molecular studies have identified genetic alterations on chromosome 11p as one of the most frequent events during the progression of bladder cancer (Habuchi et al, 1993; Shaw and Knowles, 1995; Voorter et al, 1996; Gibas and Gibas, 1997). Deletion mapping has revealed a common region of deletion between *D11*S922 (11p15.5) and *D11*S569 (11p15.1–11p15.2), but it is not yet known which particular gene constitutes the relevant target. This region contains several imprinted genes. Among these, *IGF2* and *H19* have already been implicated in urothelial carcinoma (Elkin et al, 1995; Cooper et al, 1996), but the *KIP2* (CDKN1C) gene from the same imprinted region is a further attractive candidate.

The *KIP2* gene is expressed from the maternal allele and encodes the p57$^{KIP2}$ protein, a member of the p21$^{CIP1}$ cyclin-dependent kinase inhibitor family inhibiting the G1 $\rightarrow$ S transition of the cell cycle (Lee et al, 1995; Matsuoka et al, 1995; Hatada et al, 1996; Matsuoka et al, 1996). Accordingly, the p57$^{KIP2}$ protein blocks cell proliferation when expressed in several types of cultured cells (Lee et al, 1995; Matsuoka et al, 1995; Reid et al, 1996). During development, it acts in concert with other CDK inhibitors to control tissue growth and development (Zhang et al, 1998). Some evidence implicates *KIP2* in Beckwith-Wiedemann syndrome (BWS), but alterations in *IGF2* have also been found (Reik et al, 1995; Lee et al, 1997; O'Keefe et al, 1997). BWS patients are predisposed to certain childhood cancers such as Wilms' tumour and rhabdomyosarcoma, in accord with the idea that p57$^{KIP2}$ might act as a tumour suppressor. Decreased expression of p57$^{KIP2}$ due to loss of the maternal allele or aberrant imprinting has indeed been reported in some Wilms' tumours (Hatada et al, 1996) and in individual cases point mutations have been found (O'Keefe et al, 1997). Northern analysis has shown p57$^{KIP2}$ to be expressed in specific adult tissues such as heart, brain, skeletal muscle, kidney, pancreas and testis, but not in lung and liver (Lee et al, 1995). Decreased expression has been reported in lung (Kondo et al, 1996) and adrenal (Liu et al, 1997) cancers. Expression in bladder cancer has not yet been studied.

The product of the *IGF2* gene, expressed from the paternal allele, acts as an autocrine or paracrine growth factor and its overexpression could therefore contribute to tumour growth. For instance, overexpression of *IGF2* through biallelic expression caused by loss of imprinting has been identified as an early change in the development of Wilms' tumours (Ogawa et al, 1993; Rainier et al, 1993; Weksberg et al, 1993; Steenman et al, 1994; Moulton et al, 1994; Taniguchi et al, 1995; Okamoto et al, 1997), but has also been observed in bladder cancer (Elkin et al, 1995). The *H19* gene, expressed from the maternal allele, encodes an RNA abundant in foetal tissues during development, but with a low level in adult tissues. The physiological role of *H19* RNA is not clear. Although tumour suppressor activity was suggested by studies on cell lines derived from Wilms' tumours (Reid et al, 1996), other results do not fit this hypothesis (Leighton et al, 1995). Overexpression of *H19* RNA as detected by in situ hybridization has been reported in high-grade invasive bladder cancer (Cooper et al, 1996).

The regulation of the genes in the imprinted region on chromosome 11p15.5 has turned out to be extremely complex. Most recently, an imprinting centre was identified within the $K_vQLT$ gene that is thought to control the expression of *KIP2* and several other genes on the centromeric side of $K_vQLT$, but not of the telomeric *H19* and *IGF2* genes (Lee et al, 1999; Smilinich et al, 1999). Therefore, altered expression of *KIP2* could not only be due to loss of the gene itself, but also to changes in distant segments of DNA on the same chromosome. In addition, loss of

*Received 10 January 2000*
*Revised 20 April 2000*
*Accepted 28 April 2000*

*Correspondence to:* WA Schulz imprinting could be caused by altered DNA methylation which is prevalent in bladder cancers (Jürgens et al, 1996). Again, this may occur at the *KIP2* locus itself or at a distinct control region (Dao et al, 1999).

Since however, for each of the three genes, deletions as well as epigenetic changes would be expected to affect their mRNA levels, we decided to study expression at this level by quantitative RT-PCR in a series of bladder carcinomas and corresponding normal tissue to elucidate which of them might represent a crucial target during bladder cancer progression.

### MATERIALS AND METHODS

### Tissue specimens

Twenty-four paired samples of tumour and normal tissue were obtained from patients undergoing radical cystectomy. From the cystectomy specimens paired samples of urothelial carcinoma and morphologically normal mucosa were identified, immediately cast in liquid nitrogen and stored at –80°C. Patient data and tumour characteristics are listed in Table 1. All tumours were transitional cell carcinomas of the urinary bladder. Grading and staging were performed according to the TNM classification (UICC).

### Cell lines

The human bladder cancer cell lines, J82, VMCubI, VMCubII, VMCubIII, T24, 647V, 5637, HT1376, RT-4, 639V, TCCsup, SW1710, 253J and BFTC909 were cultured as described previously (Grimm et al, 1995).

### RNA extraction

Total RNA was prepared from pre-confluent cell monolayers or frozen tissue by guanidinium/acid phenol/chloroform extraction (TRIzolReagent, Life Technologies, Berlin, Germany) as suggested by the supplier. Following re-extraction with chloroform and precipitation with isopropanol, RNA was re-dissolved in diethyl pyrocarbonate-treated water and quantified by spectrophotometry.

### Reverse transcription and PCR

Quantitative analysis of mRNA levels was performed essentially as described (Clasen et al, 1998; Oya et al, 1998). Primer sequences and amplification conditions are listed in Table 2. For quantitative analysis, glyceraldehyde-3-phosphate dehydrogenase (GAPDH) mRNA was co-amplified as an internal control and each reaction was performed within the linear phase of amplification. Each reaction was begun with an initial cycle of 5 min denaturation at 96°C, 5 min at the specific annealing temperature, and 90 s extension at 72°C, followed by the indicated number of definitive cycles of 96°C for 30 s, the specific annealing temperature for 45 s, and 72°C for 90 s. A final extension was performed at 72°C for 10 min. PCR products were separated on a 2% agarose gel before overnight transfer to a nylon membrane (Hybond-N+, Amersham, UK). Following detection with alkaline phosphatase-conjugated anti-digoxigenin antibody (Boehringer-Mannheim, Germany) luminescence signals were quantified from appropriately exposed films by video densitometry (ONE-D-SCAN 1.0, Scanalytics, MA). Values were related to GAPDH and are expressed as arbitrary expression units (AU) calculated as each gene signal/GAPDH signal. At least two independent measurements were performed for each sample and at least four for all deviating from the normal value. Analyses for intragenic polymorphisms were performed as described (Tadokoro et al, 1991).

### DNA extraction and LOH analysis

DNA was extracted and characterized from powdered frozen tissues or from leukocytes as described (Jürgens et al, 1996; Schulz et al, 1997). Two microsatellite markers, *D11*S1318 and *D11*S922, were used for LOH analysis. Primer sequences were obtained from the Genome Data Base. Each sense primer was marked with IRD-800 at the 5′-nucleotide. PCR was carried out in 50 μl reactions using 100 ng DNA each from tumours and corresponding leukocytes. Reaction conditions were 95°C for 5 min, followed by 32 cycles of 94°C for 1 min, 61°C (*D11*S1318) or 56°C (*D11*S922) for 1 min and 72°C for 2 min. The final extension was at 72°C for 10 min. One μl of diluted PCR product was loaded on 6% DNA sequencing gels in 1 × TBE buffer on a LI-COR automated sequencer. The ratio of alleles was calculated for each pair of normal and tumour samples using ONE-D-SCAN 1.0 software (Scanalytics, MA, USA) following the procedure described by An et al (1996).

### RESULTS

### $p57^{KIP2}$ mRNA expression in bladder cancer and cell lines

Expression of $p57^{KIP2}$ mRNA relative to GAPDH mRNA as measured by quantitative RT-PCR (Table 1, Figure 1) was diminished in bladder cancer tissues compared to normal mucosa: 0.82 ± 0.49 vs 1.24 ± 0.58 AU ($n$ = 24), but the difference was not statistically significant. Expression in normal mucosa ranged from 0.57–2.87 AU and in tumours from 0.01–1.62 AU. Overexpression defined as a more than two-fold increase over the level found in corresponding normal mucosa was neither observed in primary tumours nor in bladder cancer cell lines. Loss of expression defined as less than 10% expression compared to normal mucosa was observed in four tumours (4/24 = 17%) and in 5/14 (29%) cell lines (Table 3). Diminished expression, defined as from 10–50% of that in corresponding normal mucosa, was observed in five patients. In six cell lines, mRNA levels from 10–50% of average normal mucosa were measured. Therefore, overall, expression of $p57^{KIP2}$ mRNA was significantly decreased in nine patients (9/24 = 38%) and in eight cell lines (11/14 = 79%). Among the tumour tissues, no association of decreased expression with tumour stage or grade was apparent. In particular, loss of expression was found in several tumours of comparatively low stage or grade (Table 1). However, in this regard the data is skewed towards invasive, high-grade tumours and needs to be considered with caution.

### *IGF2* mRNA expression in bladder cancer and in bladder cancer cell lines

The average expression level of IGF2 mRNA relative to GAPDH mRNA (Table 1, Figure 1) was almost identical between cancer tissues and normal mucosa: 1.18 ± 1.27 vs 1.19 ± 0.63 AU ($n$ = 24).

© 2000 Cancer Research Campaign

**Table 1** Expression of 11p15.5 genes in bladder carcinomas (**A**) Clinical data, (**B**) results

| No | Age | Sex | Tumour Stage | Lymph node status | Tumour grade |
|---|---|---|---|---|---|
| 1 | 57 | M | pT2 | pN0 | 2 |
| 2 | 62 | M | pT3a | pN0 | 2 |
| 3 | 60 | F | pT3b | pN2 | 3 |
| 4 | 60 | M | pT3b | pN2 | 3 |
| 5 | 60 | M | pT1 | pN0 | 2 |
| 6 | 74 | M | pT3b | pN0 | 2 |
| 7 | 68 | F | pT3a | pN0 | 3 |
| 8 | 78 | M | pT4 | pN1 | 3 |
| 9 | 77 | M | pT3b | pN0 | 3 |
| 10 | 61 | M | pT1 | pN0 | 3 |
| 11 | 66 | F | pT1 | pN0 | 3 |
| 12 | 72 | M | pT2 | pN0 | 3 |
| 13 | 73 | F | pT4 | pN2 | 3 |
| 14 | 56 | M | pT2 | pN0 | 3 |
| 15 | 59 | M | pT4 | pN0 | 3 |
| 16 | 70 | M | pTa | pN0 | 2 |
| 17 | 60 | M | pT3b | pN0 | 3 |
| 18 | 66 | M | pT3b | pN2 | 3 |
| 19 | 75 | F | pT2 | pN0 | 2 |
| 20 | 66 | M | pTa | pN0 | 2 |
| 21 | 69 | M | pT2 | pN0 | 3 |
| 22 | 70 | M | pT2 | pN0 | 3 |
| 23 | 65 | M | pT2 | pNx | 3 |
| 24 | 64 | M | pT3b | pN1 | 3 |

| No | p57 | | | IGF-II | | | H19 | | | LOH | |
|---|---|---|---|---|---|---|---|---|---|---|---|
| | N | T | T:N | N | T | T:N | N | T | T:N | D11S922 | D11S1318 |
| 1 | 1.93 | 1.34 | 0.69 | 1.20 | 1.05 | 0.88 | 1.60 | 1.11 | 0.69 | NI | RET |
| 2 | 0.97 | 1.25 | 1.29 | 1.90 | 1.67 | 0.88 | 1.37 | 1.60 | 1.17 | RET | RET |
| 3 | **2.87** | **0.88** | **0.31** | 1.22 | 2.03 | 1.66 | 1.61 | 1.21 | 0.75 | MI | RET |
| 4 | **0.94** | **0.02** | **0.02** | 0.63 | 1.06 | 1.68 | **0.95** | **0.05** | **0.05** | n.d. | n.d. |
| 5 | 0.95 | 0.58 | 0.59 | **1.79** | **0.50** | **0.33** | 0.94 | 1.10 | 1.17 | NI | LOH |
| 6 | 1.10 | 1.25 | 1.14 | 1.79 | 1.51 | 0.84 | 1.12 | 1.10 | 0.98 | RET | RET |
| 7 | 1.88 | 0.97 | 0.52 | 1.17 | 1.62 | 1.38 | 1.50 | 0.85 | 0.57 | NI | LOH |
| 8 | 1.13 | 1.46 | 1.29 | 0.49 | 0.45 | 0.92 | 1.34 | 1.35 | 1.01 | NI | NI |
| 9 | **1.33** | **0.04** | **0.03** | **1.23** | **0.01** | **0.01** | 1.29 | 2.17 | 1.68 | RET | LOH |
| 10 | 0.69 | 0.73 | 1.06 | 1.08 | 1.30 | 1.20 | 0.96 | 0.61 | 0.64 | n.d. | n.d. |
| 11 | **0.90** | **0.07** | **0.08** | 0.90 | 0.68 | 0.76 | **0.78** | **0.01** | **0.01** | NI | NI |
| 12 | 1.00 | 1.01 | 1.01 | 0.48 | 0.44 | 0.92 | 1.30 | 0.98 | 0.75 | n.d. | n.d. |
| 13 | **1.01** | **0.41** | **0.41** | 0.50 | 0.47 | 0.94 | 0.42 | 0.22 | 0.52 | n.d. | n.d. |
| 14 | 1.24 | 1.61 | 1.30 | 0.51 | 0.42 | 0.82 | 1.12 | 1.70 | 1.52 | RET | NI |
| 15 | 1.35 | 1.20 | 0.96 | 1.65 | 1.11 | 0.67 | 1.75 | 0.90 | 0.51 | RET | RET |
| 16 | **0.60** | **0.01** | **0.02** | **1.05** | **0.18** | **0.17** | **0.85** | **0.04** | **0.04** | LOH | LOH |
| 17 | **2.55** | **1.17** | **0.46** | **2.12** | **0.91** | **0.43** | 1.25 | 1.28 | 0.89 | RET | NI |
| 18 | 0.87 | 0.81 | 0.93 | 1.11 | 1.25 | 1.13 | 0.74 | 0.99 | 1.34 | RET | RET |
| 19 | **0.96** | **0.40** | **0.42** | 0.71 | 1.04 | 1.46 | ***0.55*** | ***2.02*** | ***3.67*** | LOH | LOH |
| 20 | 0.69 | 0.57 | 0.83 | 0.77 | 1.33 | 1.82 | 1.15 | 1.18 | 1.03 | LOH | NI |
| 21 | 0.57 | 0.60 | 1.05 | 0.91 | 1.00 | 1.10 | 1.09 | 1.29 | 1.18 | RET | LOH |
| 22 | 1.44 | 1.02 | 0.71 | **0.79** | **0.03** | **0.04** | 0.90 | 0.84 | 0.93 | RET | RET |
| 23 | 1.24 | 1.62 | 1.31 | 1.33 | 1.76 | 1.32 | 0.95 | 0.98 | 1.03 | n.d. | n.d. |
| 24 | **1.58** | **0.74** | **0.47** | ***3.17*** | ***6.53*** | ***2.06*** | 1.04 | 1.16 | 1.12 | RET | RET |

N, T: mRNA expression (AU) in normal and tumour tissue, respectively; T:N quotient: bold letters mark deviating samples, italics those with overexpression. LOH: results of LOH analysis, LOH = loss of heterozygosity; RET: retention; NI: not informative; MI: microsatellite instability; n.d.: not done because DNA was unavailable

Expression ranged from 0.48–2.12 AU in normal mucosa and from 0.01–2.03 in cancer tissue. In one bladder tumour (#24) and in the corresponding morphologically normal mucosa, IGF2 mRNA was found to be overexpressed 5.5-fold and 2.7-fold, respectively. This patient was heterozygous at the Apa I site in the *IGF2* gene, and expression was found to be biallelic in tumour as well as in mucosa tissue. Overexpression of *IGF2* mRNA also occurred in the cell lines T24 and HT1376 (Table 3). Loss of *IGF2* mRNA expression was observed in two patients (2/24 = 8%) and in eight cell lines (8/14 = 57%). Diminished expression was observed in three patients and in one cell line (Figure 1). Overall, *IGF2* mRNA expression was decreased in five patients (5/24 = 21%) and in nine cell lines (9/14 = 64%). No correlation of altered *IGF2* expression with tumour stage or grade was evident.

 *© 2000 Cancer Research Campaign*

**Table 2** PCR conditions used

| Gene | Sequence of primers (sense/antisense) | Annealing temperature (°C) | Cycles | Amplicon size (bps) | Extras |
|---|---|---|---|---|---|
| *p57* | 5′-GCGGCGATCAAGAAGCTGTC-3′<br>5′-CCGGTTGCTGCTACATGAAC-3′ | 60 | 27 | 269 | hot start<br>6% DMSO |
| *IGF-II* | 5′-CATTGCTTACCGCCCCAG-3′<br>5′-AGTACGTCTCCAGGAGGGCC-3′ | 62 | 23 | 200 | 2% formamide |
| *H19* | 5′-TACAACCACTGCACTACCTG-3′<br>5′-TGGAATGCTTGAAGGCTGCT-3′ | 61 | 21 | 575 | 3% formamide |
| *GAPDH* | 5′-TCCCATCACCATCTTCCA-3′<br>5′-CATCACGCCACAGTTTCC-3′ | 60–62 | 17 | 380 | accordingly<br>as above |

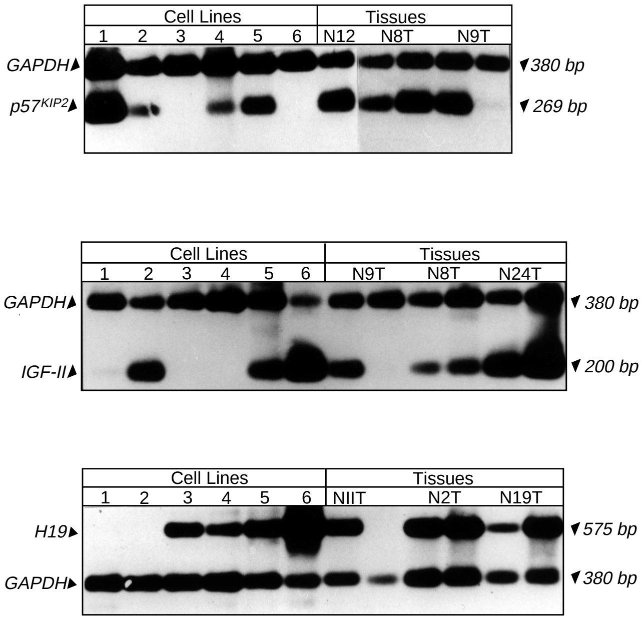

**Figure 1** Expression of 11p15.5 genes in urothelial carcinoma. The figure shows typical luminographs from quantitative RT-PCR analyses of $p57^{KIP2}$ (top), *IGF-II* (middle), and *H19* (bottom) mRNA levels in cell lines (left) and normal (N) or urothelial tumour (T) tissues (right). GAPDH mRNA was used for comparison throughout all experiments. The sizes of the PCR products are indicated on the right-hand side. The cell lines used were: (1) VMCub III; (2) T24; (3) J82; (4) 647V; (5) 5637; (6) HT1376. The indicated tissue numbers correspond to those in Table 1. Note that the top panel displays results from two separate experiments (as evident from the different backgrounds)

### *H19* RNA expression in bladder cancer and in bladder cancer cell lines

Average expression of *H19* RNA relative to GAPDH mRNA (Table 1, Figure 1) was very similar in cancer tissues and normal mucosa: 1.03 ± 0.56 vs 1.11 ± 0.33 AU ($n$ = 24). Expression ranged from 0.42–1.75 AU in normal mucosa and from 0.01–2.17 AU in tumours. Moderate overexpression compared to the corresponding normal mucosa was observed in one bladder cancer specimen (#19). The patient was unfortunately homozygous for the intragenic Rsa I polymorphism. The cell line HT1376 also displayed an increased level of *H19* RNA (Table 3). Loss of *H19* mRNA expression (cf. Figure 1) was observed in only three tumours (3/24 = 13%) of different stages and grades, but in seven cell lines (7/14 = 50%). No tumours with a level of expression between 10–50% of that in normal mucosa were observed, but the two cell lines VMCubI and 639V showed expression levels in this range.

### Correlation of expression changes

All three patients with loss of *H19* RNA expression had concomitantly lost $p57^{KIP2}$ mRNA expression whereas decreased expression of $p57^{KIP2}$ mRNA was observed more frequently without concomitant changes in *H19*. The two tumours overexpressing either *H19* or *IGF2* mRNA had diminished expression of $p57^{KIP2}$ mRNA. Likewise, the cell line HT1376 overexpressing *IGF2* and *H19* mRNAs displayed loss of $p57^{KIP2}$ expression and the cell line T24, with a two-fold overexpression of IGF2 mRNA, showed decreased expression of $p57^{KIP2}$ mRNA. Among the five patients with decreased expression of IGF2 mRNA, three had concomitantly decreased $p57^{KIP2}$ mRNA, but in two of them it was the sole alteration. Among the cell lines, the former situation was rather frequent, being present, e.g. in J82, VmCub1, and 639V, whereas an isolated decrease in *IGF2* mRNA was found only in the black-foot disease cell line BFTC909. Notably, decreased expression of *IGF2* mRNA was not associated with increased expression of *H19* RNA or vice versa in either primary tumours or cell lines. Among the nine tumours with decreased $p57^{KIP2}$ mRNA levels, six displayed a concomitant decrease of *IGF2* and/or *H19* expression, but three patients showed this as the only alteration. Of note, one papillary tumour and the papillary tumour RT4 cell line as well as the cell line TCCsup showed an almost complete loss of expression of all three mRNAs.

### Loss of heterozygosity

Loss of heterozygosity (LOH) analysis was performed to estimate its frequency in the samples investigated. DNA suitable for LOH analysis was available from 20 of the 24 tumours and corresponding leukocytes. Two microsatellites on chromosome 11p15.5, *D11*S1318 located between the *KIP2* and *IGF2* loci and *D11*S928 telomeric to the *H19* locus, were investigated. Eighteen tumours were informative for at least one marker, seven of which (39%) showed loss of heterozygosity at one locus (Table 1). LOH was detected in 4/14 (28%) informative samples at *D11*S1318 and in 4/12 (33%) informative samples at *D11*S922, respectively. One tumour DNA (#3) yielded additional bands at *D11*S1318 suggestive of microsatellite instability. Four instances of LOH (tumours #5, 9, 16, 19) were associated with expression changes in at least one of the genes, one tumour (#7) with LOH displayed borderline expression of *KIP2* and *H19*, but two instances (#20, #21) occurred in tumours with evidently normal expression of all three genes.

© 2000 Cancer Research Campaign

**Table 3** Expression of 11p15.5 genes in bladder cancer cell lines

| Cell line | Gene *p57* | *IGF-II* | *H19* |
|---|---|---|---|
| VmCubIII | 1.21 | **0.32** | **0.01** |
| T24 | **0.41** | *2.10* | **0.06** |
| J82 | **0.01** | **0.01** | 0.81 |
| 647V | **0.30** | **0.01** | 0.62 |
| 5637 | 0.65 | 1.12 | 1.33 |
| HT1376 | **0.01** | *9.04* | *3.70* |
| VmCubI | **0.06** | **0.01** | **0.50** |
| RT4 | **0.02** | **0.03** | **0.01** |
| VmCubII | **0.47** | 0.80 | **0.03** |
| 639V | **0.13** | **0.01** | **0.51** |
| TCC-sup | **0.04** | **0.05** | **0.01** |
| SW1710 | **0.43** | **0.05** | **0.02** |
| 253J | **0.14** | 0.78 | **0.08** |
| BFTC909 | 0.76 | **0.02** | 1.52 |
| Normal Mucosa | 1.24 ± 0.58 | 1.19 ± 0.63 | 1.11 ± 0.33 |
| < 50% Mucosa | **< 0.62** | **< 0.60** | **< 0.56** |
| < 10% Mucosa | **< 0.12** | **< 0.12** | **< 0.11** |

Expression of the indicated genes relative to GAPDH is given in arbitrary units. Bold letters mark expression levels below 50% of normal mucosa, italics those with overexpression.

### DISCUSSION

The central issue addressed in this study was whether the *KIP2* gene might be a target of genetic and/or epigenetic alterations on chromosome 11p15.5 in progressive bladder cancers. Since either deletions, altered DNA methylation or loss of imprinting would be expected to result in altered levels of mRNA, we approached this question by comparing mRNA levels in paired tumour and normal mucosa samples. Indeed, more than one third of the primary tumours in our study (9/24 = 38%) and even more of the bladder cancer cell lines (11/14 = 79%) showed substantially decreased $p57^{KIP2}$ mRNA expression. Importantly, several specimens displayed complete loss of expression. Previous studies have consistently estimated the frequency of LOH on chromosome 11p15.5 in advanced bladder cancers as being 30–40%. The frequency of LOH in the present study (at least 33%) was within this usual range. Thus, the observed frequency of altered $p57^{KIP2}$ expression was that expected for a tumour suppressor gene targeted by alterations in the chromosome 11p15.5 region. It remains possible that in addition to decreased expression, point mutations contribute to loss of $p57^{KIP2}$ function in bladder cancers, even though no mutations were found in 20 tumours studied by Tokino et al (1996).

Two further imprinted genes from the same chromosomal region, *IGF2* and *H19*, are thought to be involved in growth regulation and have already been shown to be overexpressed in some urothelial carcinomas (Elkin et al, 1995; Cooper et al, 1996). Altered expression of these two genes was less frequent in primary tumours, but also highly prevalent in bladder tumour cell lines. It is important to note that with our method both tumours with overexpression and decreased expression of *IGF2* or *H19* mRNA were observed. In contrast, expression of *KIP2* was never increased. Significantly, loss of *H19* expression was found in primary tumours only in association with loss of *KIP2* expression. Since *H19* RNA is maternally expressed like $p57^{KIP2}$, the concomitant loss of expression of both genes in several tumours could be due to deletions affecting the maternal chromosome. Therefore, loss of *H19* gene expression may be secondary to loss of *KIP2*.

Nevertheless, decreased expression of *KIP2* is certainly only one among several consequences of alterations involving the chromosome 11p15.5 region. Two primary tumours (#5 and 22) and one cell line (647V) showed substantially decreased expression of *IGF2* mRNA associated with borderline (#5) or normal expression (#22) of $p57^{KIP2}$ mRNA. Several tumours, and less surprisingly most cell lines, presented multiple changes suggesting involvement of the chromosomes 11 from both parents. For instance, decreased expression of $p57^{KIP2}$ as well as *IGF2* mRNAs was found repeatedly (#9, #17, J82, VMCubI and 639V), occasionally accompanied by loss of *H19* expression as well (#16, RT-4 and TCCsup). Our study also confirms that biallelic expression of *IGF2* occurs in bladder cancer. In one case it was observed in both tumour and morphologically normal mucosa (patient #24) and may represent an early alteration as suggested (Elkin et al, 1995).

While overexpression of *IGF2* has been identified in many different cancers (McCann et al, 1996; Mori et al, 1996; Uyeno et al, 1996; Nonomura et al, 1997; Oda et al, 1998) and could therefore also contribute to bladder cancer development, it is difficult to conceive how loss of its expression might have the same effect. Nevertheless, loss of *IGF2* expression in tumour tissues and cell lines indicates that not only expression from the maternal, but also the paternal chromosome is disturbed in advanced bladder cancers. Moreover, almost none of the bladder carcinoma cell lines showed an expression pattern comparable to normal mucosa. Therefore, our data are best explained by assuming that the *KIP2* gene is an important target of genetic or epigenetic alterations on chromosome 11p15.5 in bladder cancer, but that during tumour progression often both chromosomes are affected. Several apparently independent changes have also been identified in this region in several other tumours (summarized in Karnik et al, 1998). It is therefore conceivable that the high frequency and variety of changes observed in this chromosomal region indicate that it is particularly susceptible to damage resulting from genomic instability in progressive tumours.

### ACKNOWLEDGEMENTS

We are grateful to the members of the Departments of Urology and Pathology who provided the tissues used in this study, to A Radzewitz for technical assistance, to A Florl for help with the LOH analyses, to Dr B Schmidt for introducing us to her method of quantitative RT-PCR, and to Drs R Ackermann, BJ Schmitz-Dräger, and J Walter for helpful discussions. This study was supported by the Deutsche Forschungsgemeinschaft and by the VERUM foundation.

### REFERENCES

An HX, Niederacher D, Picard F, van Roeyen C, Bender HG and Beckmann MW (1996) Frequent allele loss on 9p21–22 defines a smallest common region in the vicinity of the CDKN2 gene in sporadic breast cancer. *Genes, Chromosomes Cancer* **17:** 14–20

Clasen S, Schulz WA, Gerharz CD, Grimm MO, Christoph F and Schmitz-Dräger BJ (1998) Frequent and heterogeneous expression of cyclin-dependent kinase inhibitor WAF1/p21 protein and mRNA in urothelial carcinoma. *Br J Cancer* **77:** 515–521

Cooper MJ, Fischer M, Komitowski D, Shevelev A, Schulze E, Ariel I, Tykocinski ML, Miron S, Ilan J, de Groot N and Hochberg A (1996) Developmentally imprinted genes as markers for bladder tumor progression. *J Urol* **155:** 120–127

Dao D, Walsh CP, Yuan L, Gorelov D, Feng L, Hensle T, Nisen P, Yamashiro DJ, Bestor TH and Tycko B (1999) Multipoint analysis of human chromosome

 © 2000 Cancer Research Campaign

11p15/mouse distal chromosome 7: inclusion of H19/IGF2 in the minimal WT2 region, gene specificity of H19 silencing in Wilms' tumorigenesis and methylation hyper-dependence of H19 imprinting. *Hum Mol Genet* **8:** 1337–1352

Elkin M, Shevelev A, Schulze E, Tykocinsky M, Cooper M, Ariel I, Pode D, Kopf E, de Groot N and Hochberg A (1995) The expression of the imprinted H19 and IGF-2 genes in human bladder carcinoma. *FEBS Lett* **374:** 57–61

Gibas Z and Gibas L (1997) Cytogenetics of bladder cancer. *Cancer Genet. Cytogenet* **95:** 108–115

Grimm MO, Jürgens B, Schulz WA, Decken K, Makri D and Schmitz-Dräger BJ (1995) Inactivation of tumor suppressor genes and deregulation of the c-myc gene in urothelial cancer cell lines. *Urol Res* **23:** 293–300

Habuchi T, Ogawa O, Kaekehi Y, Ogura K, Koshiba M, Hamazaki S, Takahashi R, Sugiyama S and Yoshida O (1993) Accumulated allelic losses in the development of invasive urothelial cancer. *Int J Cancer* **53:** 579–584

Hatada I, Inazawa J, Abe T, Nakayama M, Kaneko Y, Jinno Y, Niikawa N, Ohashi H, Fukushima Y, Iida K, Yutani C, Takahashi S, Chiba Y, Ohishi S and Mukai T (1996) Genomic imprinting of human $p57^{KIP2}$ and its reduced expression Wilms' tumors. *Human Mol Genet* **5:** 783–788

Jürgens B, Schmitz-Dräger BJ and Schulz WA (1996) Hypomethylation of L1 LINE sequences prevailing in human urothelial carcinoma. *Cancer Res* **56:** 5698–5703

Karnik P, Chen P, Paris M, Yeger H and Williams BRG (1998) Loss of heterozygosity at chromosome 11p15 in Wilms tumors: identification of two independent regions. *Oncogene* **17:** 237–240

Kondo M, Matsuoka S, Uchida K, Osada H, Nagatake M, Takagi K, Harper JW, Takahashi T, Elledge SJ and Takahashi T (1996) Selective maternal-allele loss in human lung cancers of the maternally expressed $p57^{KIP2}$ gene at 11p15.5. *Oncogene* **12:** 1365–1368

Lee M-H, Reynisdóttir I and Massagué J (1995) Cloning of $p57^{KIP2}$, a cyclin-dependent kinase inhibitor with unique domain structure and tissue distribution. *Genes Dev* **9:** 639–649

Lee MP, DeBaun M, Randhawa G, Reichard BA, Elledge SJ and Feinberg AP (1997) Low frequency of $p57^{KIP2}$ mutations in Beckwith-Wiedemann syndrome. *Am J Hum Genet* **61:** 304–309

Lee MP, DeBaun M, Mitsuya K, Galonek HL, Brandenburg S, Oshimura M and Feinberg AP (1999) Loss of imprinting of a paternally expressed transcript, with antisense orientation to $K_v$QLTI, occurs frequently in Beckwith-Wiedemann syndrome and is independent of insulin-like growth factor II imprinting. *Proc Natl Acad Sci USA* **96:** 5203–5208

Leighton PA, Ingram RS, Eggenschwiler J, Efstratiadis A and Tilghman SM (1995) Disruption of imprinting caused by deletion of the H19 gene in mice. *Nature* **375:** 34–39

Liu J, Kahri AI, Heikkila P and Voutilainen R (1997) Ribonucleic acid expression of the clustered imprinted genes, $p57^{KIP2}$, insulin-like growth factor II, and H19, in adrenal tumors and cultured adrenal cells. *J Clin Endocrinol Metab* **82:** 1766–1771

Matsuoka S, Edwards MC, Bai C, Parker S, Zhang P, Baldini A, Harper JW and Elledge SJ (1995) $P57^{KIP2}$, a structurally distinct member of the $p21^{CIP1}$ CDK inhibitor family, is a candidate tumor suppressor gene. *Genes Dev* **9:** 650–662

Matsuoka S, Thompson JS, Edwards MC, Barletta JM, Grundy P, Kalikin LM, Harper JW, Elledge SJ and Feinberg AP (1996) Imprinting of the gene encoding a human cyclin-dependent kinase inhibitor, on chromosome 11p15. *Proc Natl Acad Sci USA* **93:** 3026–3030

McCann AH, Miller N, O'Meara A, Pedersen I, Keogh K, Gorey T and Dervan PA (1996) Biallelic expression of the IGF2 gene in human breast disease. *Hum Mol Genet* **5:** 1123–1127

Mori M, Inoue H, Shiraishi T, Mimori K, Shibuta K, Nakashima H, Mafune K, Tanaka Y, Ueo H, Barnard GF, Sugimachi K and Akiyoshi T (1996) Relaxation of insulin-like growth factor 2 gene imprinting in esophageal cancer. *Int J Cancer* **68:** 441–446

Moulton T, Crenshaw T, Hao Y, Moosikasuwan J, Lin N, Dembitzer F, Hensle T, Weiss L, McMorrow L, Loew T, Kraus W, Gerad W and Tycko B (1994) Epigenetic lesions at the H19 locus in Wilms' tumour patients. *Nat Genet* **7:** 440–447

Nonomura N, Nishimura K, Miki T, Kanno N, Kojima Y, Yokoyama M and Okuyama A (1997) Loss of imprinting of the insulin-like growth factor II gene in renal cell carcinoma. *Cancer Res* **57:** 2575–2577

Oda H, Kume H, Shimizu Y, Inoue T and Ishikawa T (1998) Loss of imprinting of IGF2 in renal cell carcinomas. *Int J Cancer* **75:** 343–346

Ogawa O, Eccles MR, Szeto J, McNoe LA, Yun K, Maw MA, Smith PJ and Reeve AE (1993) Relaxation of insulin-like growth factor II gene imprinting implicated in Wilms' tumour. *Nature* **362:** 749–752

Okamoto K, Morrison IM, Taniguchi T and Reeve AE (1997) Epigenetic changes at the insulin-like growth factor II/H19 locus in developing kidney is an early event in Wilms tumorigenesis. *Proc Natl Acad Sci USA* **94:** 5367–5371

O'Keefe D, Dao D, Zhao L, Sanderson R, Warburton D, Weiss L, Anyane-Yeboa K and Tycko B (1997) Coding mutations in $p57^{KIP2}$ are present in some cases of Beckwith-Wiedmann syndrome but are rare or absent in Wilms tumors. *Am J Hum Genet* **61:** 295–303

Oya M, Schmidt B, Schmitz-Dräger BJ and Schulz WA (1998) Expression of G1 $\rightarrow$S transition regulatory molecules in human urothelial cancer. *Jap J Cancer Res* **89:** 719–726

Rainier S, Johnson LA, Dobry CJ, Ping AJ, Grundy PE and Feinberg AP (1993) Relaxation of imprinted genes in human cancer. *Nature* **362:** 747–755

Reid LH, Crider-Miller SJ, West A, Lee M-H, Massagué J and Weissman BE (1996) Genomic organization of the human $p57^{KIP2}$ gene and its analysis in the G401 Wilms' tumor assay. *Cancer Res* **56:** 1214–1218

Reik W, Brown KW, Schneid H, Le Bouc Y, Bickmore W and Maher ER (1995) Imprinting mutations in the Beckwith-Wiedemann syndrome suggested by an altered imprinting pattern in the IGF2-H19 domain. *Hum Mol Genet* **4:** 2379–2385

Schulz WA, Krummeck A, Rösinger I, Eickelmann P, Neuhaus C, Ebert T, Schmitz-Dräger BJ and Sies H (1997) Increased frequency of a null-allele for NAD(P)H:quinone oxidoreductase in patients with urological malignancies. *Pharmacogenetics* **7:** 235–239

Shaw ME and Knowles MA (1995) Deletion mapping of chromosome 11 in carcinoma of the bladder. *Genes Chromosomes Cancer* **13:** 1–8

Smilinich NJ, Day CD, Fitzpatrick GV, Caldwell GM, Lossie AC, Cooper PR, Smallwood AC, Joyce JA, Schofield PN, Reik W, Nicholls RD, Weksberg R, Driscoll DS, Maher ES, Shows TB and Higgins MJ (1999) A maternally methylated CpG island in KrLQT1 is associated with an antisense paternal transcript and loss of imprinting in Beckwith-Wiedmann Syndrome. *Proc Natl Acad Sci USA* **96**: 8664–8069

Steenman MJ, Rainier S, Dobry CJ, Grundy P, Horon IL and Feinberg AP (1994) Loss of imprinting of IGF2 is linked to reduced expression and abnormal methylation of H19 in Wilms' tumor. *Nat Genet* **7:** 433–439

Tadokoro K, Fujii H, Inoue T and Yamada M (1991) Polymerase chain reaction (PCR) for detection of ApaI polymorphism at the insulin like growth factor II gene (IGF2). *Nucleic Acids Res* **19:** 6967

Taniguchi T, Sullivan MJ, Ogawa O and Reeve AE (1995) Epigenetic changes encompassing the IGF2/H19 locus associated with relaxation of IGF2 imprinting and silencing of H19 in Wilms' tumor. *Proc Natl Acad Sci USA* **92:** 2159–2163

Tokino T, Urano T, Furuhata T, Matsushima M, Miyatsu T, Sasaki S and Nakamura Y (1996) Characterization of the human $p57^{KIP2}$ gene: alternative splicing, insertion/deletion polymorphisms in VNTR sequences in the coding region, and mutational analysis. *Hum Gen* **97:** 625–631

Uyeno S, Aoki Y, Nata M, Sagisaka K, Kayama T, Yoshimoto T and Ono T (1996) IGF2 but not H19 shows loss of imprinting in human glioma. *Cancer Res* **56:** 5356–5359

Voorter CEM, Ummelen MIJ, Ramaekers FSC and Hopman AHN (1996) Loss of chromosome 11 and 11 p/q imbalances in bladder cancer detected by fluorescence in situ hybridization. *Int J Cancer* **65:** 301–307

Weksberg R, Shen DR, Fei YL, Song QL and Squire J (1993) Disruption of insulin-like growth factor 2 imprinting in Beckwith-Wiedemann syndrome. *Nat Genet* **5:** 143–150

Zhang P, Wong C, De Pinho RA, Harper JW and Elledge SJ (1998) Cooperation between the Cdk inhibitors $p27^{KIP1}$ and $p57^{KIP2}$ in the control of tissue growth and development. *Genes & Dev* **12:** 3162–3167

*© 2000 Cancer Research Campaign*
